# Dynamic Monitoring of the Mechanical Properties of Tobacco Cells Under Salt Stress by Double Resonator Piezoelectric Cytometry

**DOI:** 10.3390/bios16040227

**Published:** 2026-04-20

**Authors:** Taomin Zhou, Tiean Zhou, Zhicheng Kong, Chengfang Tan, Weisong Pan

**Affiliations:** 1 College of Bioscience and Biotechnology, Hunan Agricultural University, Changsha 410128, Chinatanchengfang@hunau.edu.cn (C.T.); 2 Hunan Engineering Research Center for Cell Mechanics and Functional Analysis, Changsha 410128, China

**Keywords:** salt stress, microfilament cytoskeleton, cells generated surface stress, cell viscoelastic index, double resonator piezoelectric cytometry

## Abstract

Soil salinity is a major abiotic stressor that constrains plant growth and development, yet the coordinated regulatory mechanisms underlying salt stress impacts on plant cell mechanical properties and the cytoskeleton remain elusive. In this study, tobacco suspension cells were employed as a model system. Combining mechanical measurements, fluorescence microscopy imaging, and bright-field morphological observation, we systematically characterized the dynamic response patterns of cell-generated surface stress (Δ*S*), cell viscoelastic index (*CVI*), microfilament cytoskeleton structure, as well as cell morphology and plasmolysis under NaCl stress ranging from 50 to 150 mmol/L. The results revealed three distinct response thresholds: 50 mmol/L NaCl treatment induced only transient Δ*S* fluctuations and mild plasmolysis, with no significant changes in *CVI* or microfilament fluorescence intensity, suggesting a safe tolerance threshold. The 75–100 mmol/L NaCl treatments triggered reversible “rise–recovery” mechanical responses in Δ*S* and *CVI*. The microfilament cytoskeleton showed minor structural adjustments, and plasmolysis increased gradually but remained reversible, defining this range as a reversible acclimation phase. The 125–150 mmol/L NaCl treatment caused an irreversible decline in Δ*S* (with a sharp instantaneous drop at 150 mmol/L). *CVI* variations diminished and stabilized after 6 h. The microfilament cytoskeleton suffered progressive disruption, as fluorescence intensity dropped to 1% of the control group at 150 mmol/L, accompanied by severe plasmolysis and protoplast shrinkage, indicating irreversible cellular damage. These findings demonstrate a concentration-dependent gradient effect of NaCl stress, highlighting tight coordination between mechanical properties, cytoskeletal integrity, and morphological adaptation. This work provides critical cytological insights into the molecular regulation of plant salt stress responses.

## 1. Introduction

In recent years, the issue of soil salinization has become increasingly severe due to natural and anthropogenic factors [1]. Salt stress has emerged as one of the most significant abiotic stresses, leading not only to arable land degradation and adverse effects on agricultural production, but also to limitations in plant natural productivity, resulting in substantial global crop losses [2]. Plants can absorb essential nutrients (e.g., K^+^) from environments with low ionic concentrations, but they are generally unable to tolerate high concentrations of Na^+^. However, under salt stress conditions, the Na^+^ concentration surrounding cells far exceeds tolerable levels, making Na^+^ toxicity one of the primary mechanisms underlying cellular damage in most salt-sensitive plant species [3,4]. When detecting changes in the external environment, plant cells dynamically integrate both endogenous and exogenous stimuli, which can induce alterations in their mechanical properties [5]. The assembly–disassembly of the cytoskeleton is well-established as a critical factor in regulating cell mechanics and is essential for plant responses to salt stress [6]. The dynamics of cytoskeletal organization are important for enhancing plant salt tolerance through various cellular activities [7,8]. These dynamics inevitably affect the forces generated by the cells and lead to changes in cell viscoelasticity [9], making the study of cytoskeletal networks under salt stress a vital area of research concerning the mechanisms of plant salt tolerance.

Plant cells respond to external signals via a continuum of the cell wall, plasma membrane and cytoskeleton [10]. When subjected to salt stress, plant cells experience modifications in their cell wall components. Specifically, glycoproteins in the cell wall undergo cross-linking with phenolic compounds due to the accumulation of reactive oxygen species, resulting in cell wall stiffening [11]. Isotropic pressure generated by vacuoles, which serves as a primary source of surface stress within plant cells, exerts pressure on the plasma membrane, potentially leading to cell rupture, while the cell wall resists the turgor pressure [10,12]. According to the research by Komis, following plasmolysis in a hyperosmotic environment, microtubules and microfilaments rapidly depolymerize and subsequently re-polymerize. This defensive mechanism enables cells to adjust to morphological changes induced by salt stress. Under low salt concentrations, microfilaments and microtubules can re-polymerize to support normal cellular architecture; however, under high salt stress, they primarily remain in a depolymerized state [13,14]. This depolymerization is not merely a consequence of cellular damage but represents an active adaptive strategy that facilitates rapid cytoskeletal remodeling [7], coordinates cellular responses to osmotic and ionic stresses [15], and alleviates mechanical tension at the cell wall-plasma membrane interface [16], thereby enhancing cell survival under stress conditions. Furthermore, the cytoskeleton plays a crucial role in regulating the thickening of secondary cell walls and is actively involved in signaling pathways related to the formation of the cell wall, thereby serving as a key regulator of cell wall architecture [17]. In summary, this continuum works in concert to regulate the mechanical functions of cells.

The methodologies for measuring cell mechanics include atomic force microscopy (AFM), micropipette aspiration technique (MPA), microfluidic technologies, and magnetic twisting cytometry (MTC) [18]. These techniques require either the adhesion of cells to substrates or direct contact with specific probes. At the cellular scale, heterogeneity within the cell can result in variability in biomechanical responses, and the forces exerted by these methods may induce cellular damage. Furthermore, these approaches are primarily applicable to individual cells and are less suited for the examination of cell populations. Quartz crystal microbalance (QCM) is a highly sensitive mass detection device based on the piezoelectric properties of quartz crystals. It operates by converting mass changes on the quartz crystal’s surface into corresponding variations in the frequency of the crystal oscillation circuit’s output signal. QCM exhibits notable characteristics, such as non-destructive, dynamic, and continuous monitoring of cell populations, and has been widely adopted for measuring cellular viscoelasticity [19,20,21]. However, conventional QCM systems, despite their real-time and non-destructive capabilities, typically provide only semi-quantitative viscoelastic indices (CVI) derived from a single resonator, lacking the ability to simultaneously quantify cell-generated surface stress (Δ*S*) and viscoelastic moduli. To address these limitations, our laboratory has developed a double resonator piezoelectric cytometry (DRPC) to simultaneously measure force and viscoelasticity in both animal and plant cells, by leveraging the distinct mechanical response patterns of AT-cut and BT-cut quartz crystals to surface loads (mass, stress, and viscoelasticity). The key innovation lies in the synergistic use of both crystal types: identical lateral stresses induce frequency shifts of equal magnitude but opposite signs in AT cut versus BT cut resonators. This allows DRPC to decouple and simultaneously quantify Δ*S* and CVI in real time on the same temporal scale. Employing this technique, we successfully monitored the dynamic mechanical characteristics of human umbilical vein endothelial cells (HUVEC) during adhesion, including variations in surface stress (Δ*S*) and viscoelastic moduli (G′ and G″) [22]. Recently, this method has been applied to assess the force and viscoelastic responses of rice cells and protoplasts under different concentrations of PEG6000 simulating drought stress [23], as well as to evaluate the salt tolerance of various rice cultivars through examining the changes in Δ*S* and cell viscoelastic index (*CVI*) [24]. This study used double resonator piezoelectric cytometry (DRPC) to monitor the effects of NaCl on the surface stress and viscoelasticity of tobacco cells in real time, while also observing morphological changes and the microfilament cytoskeleton via fluorescence microscopy. By quantitatively linking mechanical responses to cytoskeletal reorganization, this work provides a novel approach to deciphering plant cell dynamics under salt stress and advances abiotic stress tolerance research.

## 2. Materials and Methods

### 2.1. Materials and Major Reagents

The Lifeact-EGFP tobacco callus tissue, consisting of laboratory-constructed tobacco cells expressing enhanced green fluorescent protein (EGFP)-labeled microfilaments [25], was provided by the Hunan Provincial Engineering Technology Research Center for Cell Mechanics and Functional Analysis (Changsha, China). A QCM-922 eight-channel quartz crystal microbalance (Seiko-EG&G Inc., Tokyo, Japan) was used for all measurements. AT-cut and BT-cut quartz crystals with a fundamental frequency of 9 MHz, coated with optically transparent indium tin oxide (ITO) electrodes, were employed in the experiments. The 9 MHz AT/BT-cut quartz crystals were procured from Hangzhou Zhongjing Electronic Technology Co., Ltd. (Hangzhou, China). ITO electrode deposition was performed by Jiaxing Huazheng Optoelectronic Technology Co., Ltd. (Jiaxing, China), and the ITO deposited crystals were mounted to crystal holders by Beijing Chenjing Electronic Co., Ltd. (Beijing, China).

**Figure 1:**
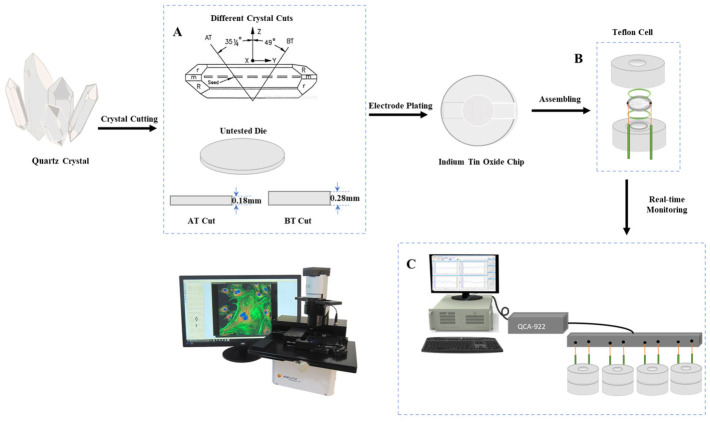
Schematic illustration of the fabrication and measurement principle of the QCM-based DRPC chip: (**A**) Schematic diagrams of AT cut and BT cut quartz crystals. AT cut and BT cut quartz crystals were sectioned at angles of 35°15′ and −49°, respectively, relative to the crystallographic *Z*-axis. Both cuts oscillate in thickness-shear mode and exhibit frequency shift responses of equal magnitude but opposite sign to the same lateral stress. (**B**) Schematic diagram of the Teflon cell assembly. (**C**) Schematic diagram of the DRPC experimental setup.

AT cut and BT cut quartz crystals were sectioned at angles of 35°15′ and −49°, respectively, relative to the crystallographic *Z*-axis. Due to the anisotropic nature of quartz, its physical properties vary along different crystallographic axes; thus, AT cut and BT cut represent distinct crystallographic orientations of quartz. Both cuts oscillate in thickness-shear mode and exhibit frequency shift responses of equal magnitude but opposite sign to the same lateral stress. Unlike traction force microscopy (TFM) and micropillars (MP), which indirectly measure cell traction forces by detecting the deformation of flexible substrates, the DRPC sensor directly quantifies cellular forces using the anisotropic properties of quartz crystals. This is achieved by exploiting the different mechanical sensitivities of Y-rotated cut quartz crystals (with varying cut angles) to cell mass, viscoelasticity, and the lateral stress exerted by cells on the crystal. The lateral stress exerted on the crystal by cells is equivalent to the traction force measured by TFM. DRPC uses AT cut and BT cut quartz resonators, with cut angles of 35°15′ and −49°, respectively. The lateral stress applied by cells to these chips affects the chip’s microstructure, altering the effective elastic constants of the quartz crystal—which determine its elastic modulus—and thus influences the crystal’s resonant frequency. The effective elastic constants are functions of the cosine and sine of the cut angle. Consequently, the lateral stress constants for AT cut and BT cut quartz crystals are nearly equal in magnitude but opposite in sign. This enables quantitative separation and measurement of cellular forces and viscoelasticity. This dual-resonator technique was first proposed by EerNisse in 1972 to measure surface stress generated by materials deposited on quartz crystals [26]. Based on this approach, the crystals used in this study were machined to the required thickness and dimensions, yielding thicknesses of 0.18 mm and 0.28 mm for the 9 MHz AT cut and BT cut crystals, respectively, as illustrated in Figure 1A. Each crystal was assembled using two silicone O-rings to form a chamber at the bottom of a polytetrafluoroethylene (PTFE) cell (Figure 1B). Additional key instruments and reagents included a Countess™ Automated Cell Counter (Invitrogen, Waltham, MA, USA), an LSM 780 Laser Scanning Confocal Microscope (Carl Zeiss, Oberkochen, Germany), a Lumascope™ 720 Fully Automated Live Cell Imaging System (Etaluma, Carlsbad, CA, USA), Poly Dimethyl Diallyl Ammonium Chloride (PDADMAC) (Sigma-Aldrich, St. Louis, MO, USA), and sodium chloride (NaCl) (Sinopharm Chemical Reagent Co., Ltd., Shanghai, China).

### 2.2. Sample Preparation

Tobacco suspension cells on the 5th day of subculture were selected as experimental materials due to their high quantity and viability. These cells were filtered through a 100-mesh cell sieve to remove larger clumped cells, and the remaining cells were collected using a 500-mesh cell sieve. Cells of uniform size and dispersion on the filter screen were then resuspended in culture media to create the cell suspension. The resulting cell concentration was (3.0 ± 0.5) × 10^5^ cells/mL.

### 2.3. Surface Modification and Pretreatment of ITO Electrode

The ITO electrode was immersed in a 2% sodium dodecyl sulfate (SDS) solution for 15 min, rinsed with absolute ethanol and deionized water, and then dried with nitrogen. This process was repeated three times to obtain a clean ITO electrode. The electrode surface was then modified by immersion in a 1% PDADMAC solution in the dark for 30 min. Excess modification solution was removed, and the electrode was dried with nitrogen. PDADMAC is a strongly cationic polyelectrolyte with non-toxicity, high water solubility, strong cohesive properties, excellent hydrolytic stability, and non-gelling characteristics. It forms a monolayer of several nanometers in thickness at interfaces, rendering it suitable as a cell adhesion promoter in this study [27]. Infrared spectroscopy confirms that the 1% PDADMAC coating exhibits a strong N–H peak at 3300–3500 cm^−1^, indicating the presence of positively charged groups. Q-sense measurements further show that cell addition leads to a decrease in frequency and an increase in dissipation, confirming effective electrostatic interaction between cells and the PDADMAC-modified surface [28]. Additionally, studies have demonstrated that a 1% PDADMAC coating forms a uniform thin film that efficiently adsorbs negatively charged tobacco BY-2 cells [29]. Together, these findings indicate that the PDADMAC-modified surface supports the formation of a relatively uniform monolayer of cells, ensuring stable and reproducible QCM measurements. All parts of the Teflon detection cell were completely soaked in a beaker containing absolute ethanol, ultrasonically cleaned for 5 min, then transferred to a beaker containing deionized water for another 5 min of ultrasonic cleaning. The parts were subsequently dried with nitrogen and placed in an ultra-clean workbench for UV irradiation for 30 min. The modified ITO electrode was then loaded into the Teflon detection cell. Finally, 600 μL of tobacco cell culture media was added and allowed to rest overnight to relieve the stress generated during the loading process.

### 2.4. Monitoring of Mechanical Properties of Tobacco Cells Under Different Concentrations of Salt Stress

The Teflon detection cell was equilibrated overnight and connected to the QCM-922 eight-channel quartz crystal microbalance system to monitor the resonance frequency (*F*) and motional resistance (*R*) in real time. After approximately 4 h of stabilization (F and R reached steady-state values), 300 μL of culture medium was carefully aspirated and replaced with 300 μL of the prepared tobacco cell suspension (Section 2.2). The system was allowed to run for an additional 4 h to ensure stable cell adhesion before NaCl treatment. To minimize volume disturbances and alterations to the cellular microenvironment, a high-concentration NaCl stock solution (5 M) was introduced in small volumes. The working volume in the measurement chamber was maintained at 600 μL. The required volumes of NaCl stock solution were calculated to achieve final target concentrations (50, 75, 100, 125, and 150 mmol/L), with each addition restricted to 6–18 μL (≤3% of the total system volume). The NaCl solution was slowly injected into the chamber using a micropipette to reduce mechanical perturbation. Following NaCl addition, real-time changes in surface stress (Δ*S*) and cellular viscoelasticity were recorded under salt stress conditions. The transient frequency (F) and resistance (R) responses observed during solution addition primarily arose from viscodensity effects (due to viscosity and density changes in the medium). However, since these effects are accounted for and eliminated in the theoretical model, the minor volume adjustments introduced negligible interference, ensuring reliable data interpretation [22].

### 2.5. Real-Time Monitoring of Microfilament Cytoskeleton in Tobacco Cells Under Salt Stress

#### 2.5.1. Live-Cell Imaging

A 1% PDADMAC solution was added to a cell well plate and incubated in the dark for 30 min. Excess solution was removed, and the plate was dried with nitrogen. The well plate was transferred to a Lumascope™ 720 fully automated live cell imaging system, and the microscope field of view was adjusted. Cell suspension (800 μL, (3.0 ± 0.5) × 10^5^ cells/mL) was added and allowed to stand for 30 min. Once cells had adhered stably to the bottom, the focal length was fine-tuned, imaging parameters were set, and the total imaging duration was configured to 8 h with a 10 min interval. NaCl solutions were then added to achieve final concentrations of 50, 75, 100, 125, and 150 mmol/L, and imaging was started. All cells used in this study were from the same batch to ensure consistency.

#### 2.5.2. Confocal Laser Scanning Microscopy

To achieve a semi-solid state, 0.6 g·L^−1^ plant gel was added to the culture medium, preventing cell movement upon NaCl addition and maintaining focal plane stability. The semi-solid medium was thoroughly mixed with resuspended cells to obtain a final concentration of (3.0 ± 0.5) × 10^5^ cells/mL. The cell suspension was transferred to a confocal dish and placed on the stage of a laser confocal microscope. After locating the desired field of view and adjusting image acquisition parameters, NaCl solutions were added to achieve final concentrations of 50, 75, 100, 125, and 150 mmol/L. Images were captured every 1–2 min over 1 h using a 60× oil-immersion lens at a resolution of 1024 × 1024 pixels.

### 2.6. Determination of Osmotic Pressure Under Different NaCl Concentrations

The freezing point osmometer was calibrated using 50 and 850 mOsm·kg^−1^ standard solutions, and its accuracy was verified with a 290 mOsm·kg^−1^ standard solution. Osmotic pressure of the experimental systems at varying NaCl concentrations was then measured. Each concentration was tested in triplicate to ensure precision and correct any deviations.

### 2.7. Calculation of Cell-Generated Surface Stress and Cell Viscoelasticity Index

When a double resonator piezoelectric cytometry (DRPC) chip is applied to monitor and adhere to plant cells, its frequency change is mainly affected by the stress (Δ*f*_s_), cell mass (Δ*f*_m_) and cell viscoelasticity (Δ*f*_visco_) generated and applied on the surface of quartz crystals. Therefore, the relative frequency change caused by the cell adhesion process can be expressed as

(1) $$\Delta f/f_{0}=\Delta f_{s}/f_{0}+\Delta f_{m}/f_{0}+\Delta f_{\mathrm{visco}}/f_{0}$$

Since the detection depth of 9 MHz acoustic frequency is far less than the cell thickness, the cell can be regarded as a semi-infinite viscoelastic load. Therefore, according to the relationships between surface stress, mass, semi-infinite viscoelastic load and frequency shift, Equation (1) can be expressed as

(2) $$\Delta f/f_{0}=K\Delta S/t_{q}-(\sqrt{\rho_{q}\mu_{q}}{)}^{-1}\left(2f_{0}\Delta m+\pi^{-1}\sqrt{\rho_{c}\left(\left|G^{*}\right|-G^{\prime }\right)/2}\right)$$

Applying Equation (2) to AT cut and BT cut, we have:

(3) $$\Delta f^{\mathrm{AT}}/f_{0}^{\mathrm{AT}}\;=\;K^{\mathrm{AT}}\Delta S/t_{q}^{\mathrm{AT}}-(\sqrt{\rho_{q}{\mu_{q}}^{\mathrm{AT}}}{)}^{-1}\left(2f_{0}^{\mathrm{AT}}\Delta m\;+\;\pi^{-1}\sqrt{\rho_{c}\left(\left|G^{*}\right|-G^{\prime }\right)/2}\right)$$

(4) $$\Delta f^{\mathrm{BT}}/f_{0}^{\mathrm{BT}}=K^{\mathrm{BT}}\Delta S/t_{q}^{\mathrm{BT}}-(\sqrt{\rho_{q}{\mu_{q}}^{\mathrm{BT}}}{)}^{-1}\left(2f_{0}^{\mathrm{BT}}\Delta m+\pi^{-1}\sqrt{\rho_{c}\left(\left|G^{*}\right|-G^{\prime }\right)/2}\right)$$

Consider the relationship between quartz crystal thickness, crystal frequency and elastic modulus:

(5) $$t_{q}\;=\;1/(2f_{0}\frac{\rho_{q}}{\mu_{q}}{)\;}^{\frac{1}{2}}$$

According to the comprehensive Equations (3)–(5), and when the crystal frequencies of AT and BT cuts are the same, i.e., $f_{0}^{\mathrm{AT}}\;$=$\;f_{0}^{\mathrm{BT}}$=$\;f_{0;}$ the mass and viscoelastic effects can be eliminated, thus the relationship of Δ*S* is obtained:

(6) $$\Delta S_{t}\;=\;f_{0}^{-1}(K^{\mathrm{AT}}-K^{\mathrm{BT}}{)}^{-1}(\Delta f_{t}^{\mathrm{AT}}t_{q}^{\mathrm{AT}}-\Delta f_{t}^{\mathrm{BT}}t_{q}^{\mathrm{BT}})$$

Here, *K* is the stress coefficient of quartz crystal, *K*^AT^ = 2.75 × 10^−l2^ cm^2^ dyn^−1^, *K*^BT^ = −2.65 × 10^−l2^ cm^2^ dyn^−1^, *t*_q_ is the thickness of the quartz crystal, and the relationship between the thickness of the two cutting types and their frequencies is determined by their respective frequency constants *N*. Therefore, for the quartz crystal with a determined frequency, its thickness *t*_q_ is also determined accordingly. The fundamental frequency of the quartz crystal used in this study is 9 MHz, *t*_q_^AT^ = 0.0185 cm, *t*_q_^BT^ = 0.0282 cm; Δ*f*_t_^AT^ and Δ*f*_t_^BT^ are the frequency shifts in AT-cut and BT-cut quartz crystals relative to their frequencies in cell culture media at any time *t*, respectively. Those constants can be substituted into (6), which can then be simplified to

(7) $$\Delta S=380.8\Delta f^{\mathrm{AT}}-582.2\Delta f^{\mathrm{BT}}$$

When the fundamental frequency of AT-cut and BT-cut crystals is the same and the surface state is the same, the surface stress generated by the cell on the crystal can be accurately measured, which is universal. When ∆*S* is negative, it indicates that the stress generated by the cell itself is compressive stress, and the cell is in a contraction state; when ∆*S* is positive, the cell itself generates tensile stress, and the cell is in a state of expansion.

After neglecting the minor mass effect, once Δ*S* is determined, the viscoelastic contribution of cells can be determined by subtracting the frequency variation due to surface stress from the total frequency shift. Then the cell viscoelastic index (*CVI*) can be calculated by using the frequency shift subtracted from the surface stress-induced frequency shift and the motional resistance change (∆*R*) by the following equation:

(8) $${\mathrm{CVI}}^{\mathrm{AT}}\;=\;{\Delta R}_{\mathrm{cells}}^{\mathrm{AT}}/{\Delta f}_{\mathrm{cells}}^{\mathrm{AT}}$$

### 2.8. Data Analysis

The ImageJ (version 1.48) software was used for semi-quantitative fluorescence analysis of the images, with the overall fluorescence intensity values subsequently imported into Origin 2018 to generate a semi-quantitative data change map. Data from the QCM-922 eight-channel quartz crystal microbalance were processed, analyzed, and plotted using Origin 2018.

## 3. Results

### 3.1. Effects of Different Concentrations of Salt Stress on the Microfilament Cytoskeleton and Cell Morphology of Tobacco Cells

**Figure 2:**
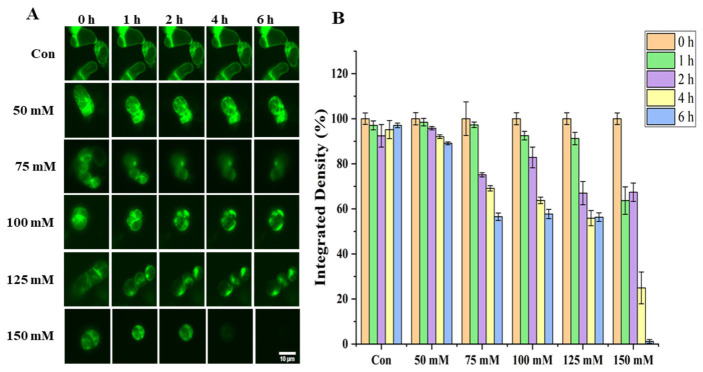
Effect of NaCl stress on the microfilament cytoskeleton of Lifeact-EGFP tobacco suspension cells: (**A**) Fluorescence images of the microfilament cytoskeleton. Scale bar: 10 µm. (**B**) Quantification of total fluorescence intensity. Data are presented as relative total fluorescence intensity. Minor variations in initial (0 h) fluorescence among groups reflect slight differences in region of interest (ROI) selection. All data were normalized to their respective 0 h baselines, revealing highly consistent temporal trends.

The fluorescence microscopy analysis (Figure 2A) demonstrated that in the control group (Con), tobacco cells exhibited a well-organized actin cytoskeleton, characterized by bright and uniform green fluorescence, indicative of intact filamentous structures. No significant alterations in fluorescence intensity or cytoskeletal morphology were observed in the 50 mmol/L NaCl-treated group compared to controls. However, exposure to 75–125 mmol/L NaCl resulted in concentration-dependent disruption of actin filaments, manifesting as progressively weaker and more diffuse fluorescence signals, along with gradual loss of structural integrity. In contrast, the 150 mmol/L NaCl group exhibited near-complete fluorescence disappearance, consistent with severe cytoskeletal disassembly.

Quantitative assessment of fluorescence integrated density (Figure 2B) revealed statistically significant (*p* < 0.05), concentration- and time-dependent alterations. Following treatment with 50 mmol/L NaCl, fluorescence intensities at all time points (1, 2, 4, and 6 h) remained largely unchanged relative to controls, confirming minimal impact on actin organization. In contrast, cells treated with 75–125 mmol/L NaCl displayed progressive fluorescence attenuation with increasing exposure duration, with more pronounced reductions at higher concentrations. After 6 h of exposure, the 125 mmol/L group exhibited an approximately 30% reduction in fluorescence intensity compared to controls, highlighting escalating structural damage with prolonged salt stress. Most strikingly, the 150 mmol/L group showed drastically diminished fluorescence at all time points, declining to 20% of control levels by 4 h and nearly 1% by 6 h, indicating rapid and irreversible cytoskeletal disassembly.

**Figure 3:**
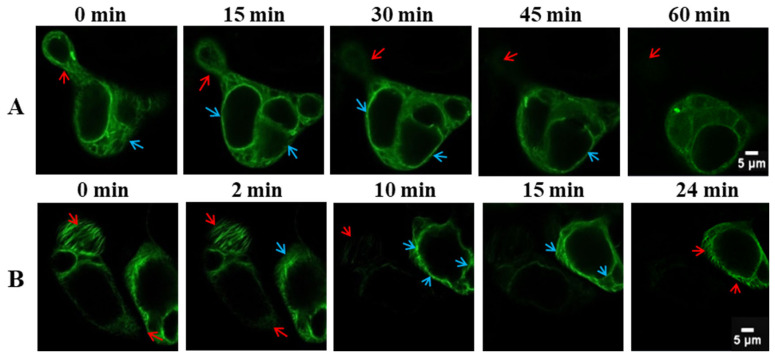
Real-time fluorescence imaging of the microfilament cytoskeleton in Lifeact-EGFP tobacco suspension cells under varying NaCl concentrations: (**A**) 100 mmol/L NaCl; (**B**) 150 mmol/L NaCl. The red arrows indicate sites of microfilament disassembly, and the blue arrows indicate sites of microfilament bundling. Scale bar: 5 µm.

Under 100 mmol/L NaCl treatment (Figure 3A), the actin cytoskeleton maintained a distribution pattern comparable to that of the control and 50 mmol/L groups. The microfilament bundles (denoted by red arrows) exhibited only minor aggregation and localized disorganization, whereas the cortical actin filaments (highlighted by blue arrows) preserved their continuous, well-defined filamentous architecture. Collectively, these findings suggest that the overall actin network retained structural integrity, indicating that this NaCl concentration induced merely subtle morphological changes without compromising cytoskeletal stability. In stark contrast, exposure to 150 mmol/L NaCl (Figure 3B) triggered rapid and profound cytoskeletal disassembly: microfilament bundles (red arrows) underwent extensive fragmentation and network disruption, accompanied by near-total disintegration of their filamentous organization. Cortical actin filaments (blue arrows) appeared markedly diffuse, with complete loss of structural continuity, while the overall fluorescence intensity experienced a precipitous decline. These dramatic morphological and fluorescent alterations unequivocally demonstrate the irreversible disassembly and functional loss of the actin cytoskeleton under severe NaCl stress.

**Figure 4:**
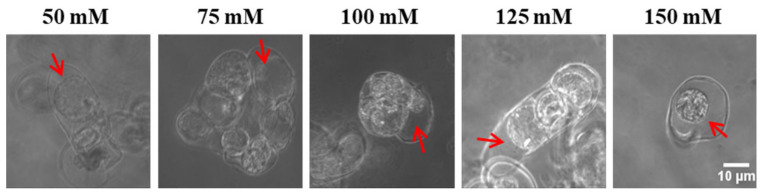
Plasmolysis of Lifeact-EGFP tobacco suspension cells 10 min after exposure to varying NaCl concentrations. Arrows indicate sites of plasmolysis (detachment of the plasma membrane from the cell wall). Scale bar: 10 µm.

Bright-field microscopy analysis of tobacco cells subjected to increasing NaCl concentrations (Figure 4) revealed distinct morphological responses across the treatment gradient. At low-to-moderate salt concentrations, cells displayed either no plasmolysis or only incipient plasmolysis, manifested as a slight separation between the plasma membrane and cell wall, while maintaining turgid, structurally intact protoplasts. This reversible phenotype implies an active cellular adaptation to osmotic stress. As NaCl concentrations escalated, cells exhibited progressively severe plasmolysis: protoplasts underwent marked contraction, accompanied by visible disorganization of subcellular architecture and clear indications of membrane damage. Under extreme salt stress, cells reached a state of terminal plasmolysis, characterized by complete protoplast-wall detachment, extreme cytoplasmic condensation, and irreversible cytomorphological deformation.

To verify the osmotic effect of NaCl treatment, the osmolality of the culture medium at different NaCl concentrations was measured using a freezing point osmometer. As shown in Table 1, the baseline osmolality of the tobacco cell culture medium was 203.67 mOsm·kg^−1^. Osmolality increased progressively with NaCl concentration, confirming that the cells were exposed to increasing osmotic stress.

### 3.2. Changes in Cell-Generated Surface Stress and Viscoelasticity of Lifeact-EGFP Tobacco Cells Under Different Concentrations of Salt Stress

**Figure 5:**
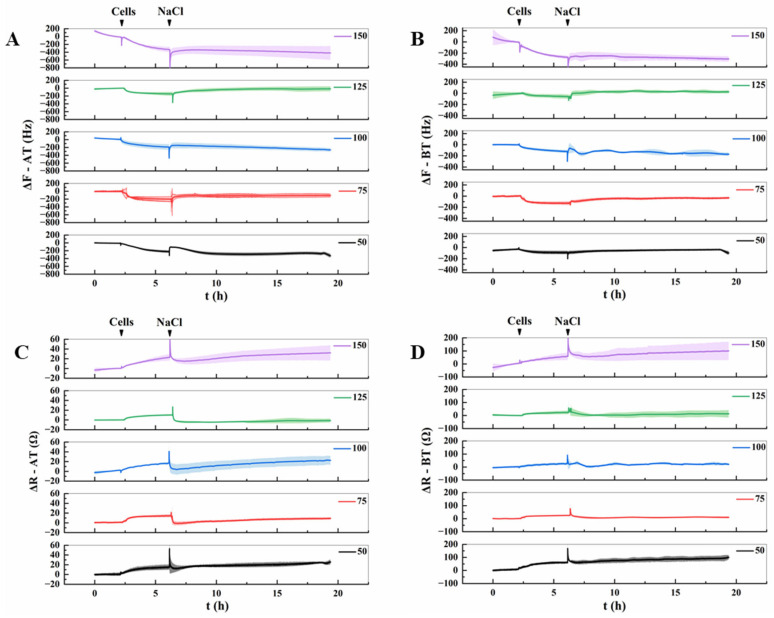
DRPC monitoring of frequency (Δ*F*) and resistance (Δ*R*) shifts induced by adhesion of Lifeact-EGFP tobacco suspension cells, followed by exposure to varying NaCl concentrations (mean ± SD, *n* = 3). Arrows indicate the time points of cell seeding and NaCl addition. (**A**,**C**): AT-cut frequency and resistance changes. (**B**,**D**): BT-cut frequency and resistance changes.

The QCM-922 eight-channel quartz crystal microbalance was employed to monitor the changes in resonance frequency (Δ*F*) and motional resistance (Δ*R*) of Lifeact-EGFP tobacco suspension cells subjected to various NaCl concentrations, as illustrated in Figure 5. After stabilization of baseline frequency (*F*) and resistance (*R*) in 600 μL of culture medium, 300 μL of cell suspension [(3.0 ± 0.5) × 10^5^ cells/mL, prepared per Section 2.2] was introduced via isovolumetric exchange. Cell adhesion to the ITO electrode surface was confirmed by characteristic QCM signatures: a sustained frequency decrease concurrent with resistance elevation, with response amplitudes varying between AT-cut and BT-cut quartz chips due to their intrinsic piezoelectric anisotropy. Following a 4 h stabilization period, the frequency and resistance in each group exhibited corresponding responses after the addition of 50, 75, 100, 125 and 150 mmol/L NaCl. The responses did not follow a linear pattern across concentrations, indicating that the mechanical properties of cells underwent distinct alterations under varying NaCl stress.

**Figure 6:**
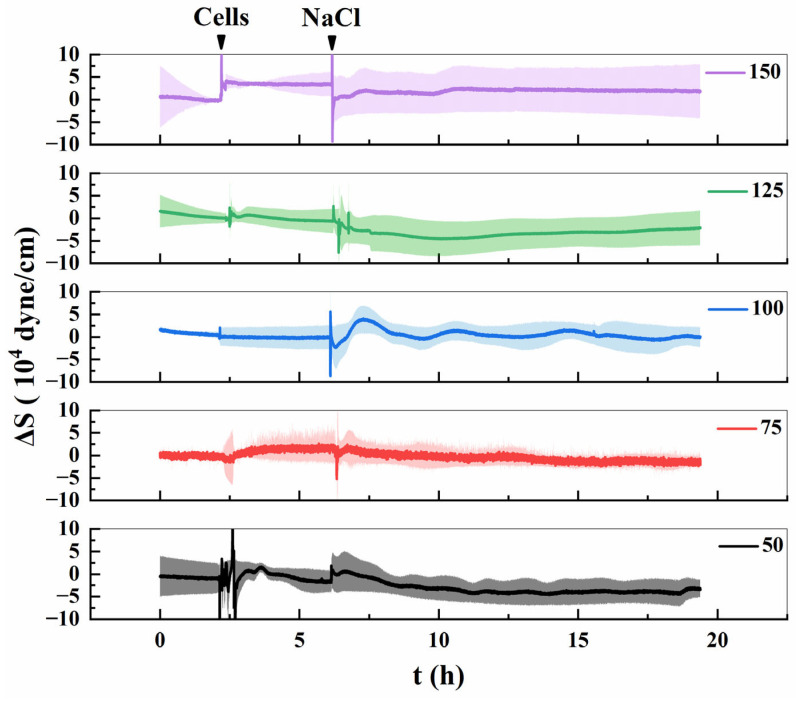
Changes in surface stress generated (Δ*S*) by Lifeact-EGFP tobacco suspension cells under different concentrations of salt stress.

Figure 6 illustrates the variations in surface stress (Δ*S*) generated by Lifeact-EGFP tobacco suspension cells under different concentrations of salt stress. The results reveal a concentration-dependent response pattern in Δ*S*. At 50 mmol/L NaCl, Δ*S* exhibited a transient, minor increase followed by a gradual decline. In contrast, the 75 mmol/L NaCl treatment did not induce significant stress elevation; Δ*S* briefly returned to baseline before decreasing steadily, remaining below initial levels throughout the observation period. Under 100 mmol/L NaCl, Δ*S* displayed a small, transient peak before stabilizing near baseline, indicating reversible behavior. At higher NaCl concentrations (125–150 mmol/L), the response pattern diverged markedly. Exposure to 125 mmol/L NaCl induced a steady decline in Δ*S* without an initial rise, stabilizing slightly below baseline. By comparison, 150 mmol/L NaCl triggered an abrupt and irreversible drop in Δ*S*, which persisted at a low level without recovery. To summarize, Δ*S* dynamics varied depending on NaCl concentration. Lower concentrations (50–100 mmol/L) produced a reversible “rise-and-fall” pattern, indicating transient cellular adaptation, whereas higher concentrations (125–150 mmol/L) resulted in a continuous and irreversible decrease, with the decline becoming more severe as NaCl levels increased. These findings align with the microfilament depolymerization and cellular responses observed in Figure 2, Figure 3 and Figure 4, further supporting the hypothesis that NaCl-induced stress disrupts cytoskeletal integrity in a concentration-dependent manner.

**Figure 7:**
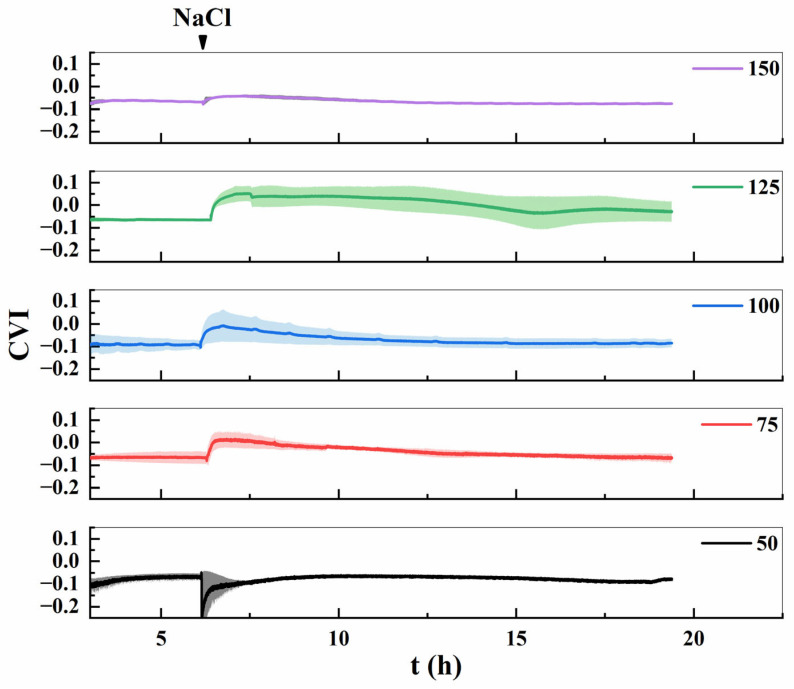
Dynamic *CVI* changes in Lifeact-EGFP tobacco suspension cells under different concentrations of salt stress.

The absolute value of the cell viscoelastic index (*CVI*) serves as an indicator of cellular mechanical properties. A larger absolute *CVI* value corresponds to softer cells with less organized internal structures, whereas a smaller absolute value reflects stiffer cells exhibiting higher structural organization [28]. As shown in Figure 7, the response patterns of *CVI* to NaCl treatment varied significantly across concentrations, closely correlating with actin filament dynamics. At 50 mmol/L NaCl, *CVI* temporarily decreased but fully recovered to baseline levels over time. This observation, consistent with the minimal microfilament disruption in Figure 2, suggests negligible inhibitory effects on cells at this concentration. Within the 75–125 mmol/L NaCl range, *CVI* initially increased from negative values toward zero, reflecting a mechanical stiffening response to salt stress, and then gradually returned to baseline as homeostasis was restored. Notably, at 150 mmol/L NaCl, *CVI* showed an attenuated rise-and-fall trend, stabilizing after 6 h (corresponding to ~12 h on the *x*-axis). This behavior aligns with the loss of fluorescence signals at 6 h (Figure 2A), indicating irreversible cellular damage under high-concentration salt stress. Across the tested range (50–125 mmol/L NaCl), cells exhibited variable viscoelastic responses, likely due to structural adaptations [30]. The stiffening effect observed at 75–125 mmol/L NaCl suggests reinforcement of cellular rigidity, potentially through cell wall modifications, as a mechanism to counteract NaCl-induced stress in Lifeact-EGFP-labeled tobacco suspension cells.

## 4. Discussion

Soil salinization imposes osmotic stress and ionic toxicity, posing major abiotic constraints on global agricultural productivity. The plant cell response to salt stress involves a temporally coordinated cascade of mechanical signaling, cytoskeletal reorganization, and morphological adaptation. However, conventional static detection techniques fail to resolve the dynamic interactions among these processes, limiting the discovery of core regulatory pathways and early-warning biomarkers. To address this gap, we employed double resonator piezoelectric cytometry (DRPC) for real-time, high-resolution tracking of cell surface stress (Δ*S*) and the cell viscoelasticity index (*CVI*) in tobacco suspension cells under NaCl stress. By integrating F-actin fluorescence imaging with morphometric analysis, this study reveals the mechanistic links between cellular mechanical adaptation and structural remodeling during salt stress.

### 4.1. Salt Stress Induces a Concentration-Dependent Threshold in Cellular Mechanical Responses

Our results indicate that the mechanical response of tobacco suspension cells to NaCl stress follows a non-linear pattern with three concentration-dependent phases. The first phase represents a safe physiological range (≤50 mmol/L), followed by a reversible adaptive phase at intermediate concentrations (75–100 mmol/L), and finally an irreversible cytotoxic range at higher concentrations (125–150 mmol/L). This triphasic behavior is evident in the trends of Δ*S* and *CVI* (Figure 5 and Figure 6) as well as in structural markers such as actin cytoskeleton integrity and the degree of plasmolysis (Figure 1, Figure 2 and Figure 3).

**Figure 8:**
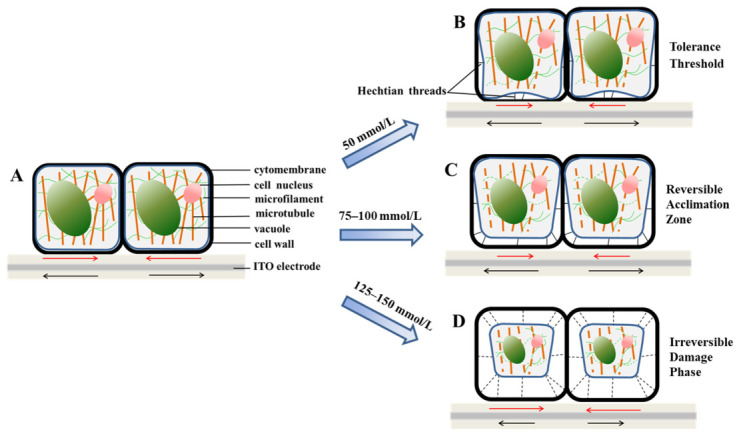
Schematic illustration of structural and mechanical responses in Lifeact-EGFP tobacco cells under NaCl stress: (**A**) Natural state: balanced surface stress. (**B**) 50 mmol/L NaCl (tolerance threshold): transient stress fluctuations, mild plasmolysis, stable *CVI* and microfilaments. (**C**) 75–100 mmol/L NaCl (reversible acclimation zone): “rise-and-recovery” Δ*S* and *CVI* response, reversible microfilament remodeling and plasmolysis. (**D**) 125–150 mmol/L NaCl (irreversible damage phase): surface stress decline (abrupt at 150 mmol/L), *CVI* attenuation, microfilament fragmentation, severe plasmolysis. Red and black arrows represent compressive stress and tensile stress respectively.

To summarize our findings, Figure 8 illustrates the dynamic changes in cellular mechanical parameters, microstructure, and morphology of transgenic Lifeact-EGFP tobacco cells in response to increasing NaCl concentrations, thereby offering insights into the role of DRPC as a biosensor for salt stress. At NaCl concentrations below 50 mmol/L (Figure 8B), cells exhibited an overall negative Δ*S* accompanied by a gradual decline in stress over time. The *CVI* transiently decreased but returned to baseline, and the actin cytoskeleton remained structurally intact, showing only dynamic rearrangement. These results suggest that low-intensity salt stress triggers mild osmotic adjustments, resulting in transient protoplast contraction without causing significant damage to internal structures. This finding is consistent with a fully tolerated cellular state. Previous studies have demonstrated that low NaCl concentrations can enhance cellular metabolism and growth in some plant systems [31,32,33,34], a trend that is also reflected at the mechanical level in our study. Liu et al. demonstrated that hypertonic stimulation increases cell traction force as cells shrink [35]. Under NaCl-induced hypertonic stress, intracellular water potential decreases, leading to cell dehydration and protoplast shrinkage, thereby exerting compressive stress on the chip, consistent with our observation of negative Δ*S* at low NaCl concentrations. The gradual stress decline likely reflects reduced water potential, which may promote water and nutrient uptake as long as *CVI* stability is maintained. When NaCl concentrations reached 75–100 mmol/L (Figure 8C), the mechanical behavior of cells shifted abruptly. Δ*S* transitioned from negative to positive, displaying a reversible rise–recovery response pattern alongside a synchronous increase in *CVI* and transient cellular stiffening. Meanwhile, the actin cytoskeleton underwent dynamic remodeling, featuring moderate bundling and depolymerization, while plasmolysis intensified yet remained reversible. These changes indicate a transition from passive osmotic response to active mechanical adaptation, implying that cells may mitigate salt-induced stress through cell wall reinforcement [11], cytoskeletal remodeling [36], and the restoration of mechanical homeostasis. During plasmolysis, the plasma membrane forms irregular inward folds, and Hechtian strands pull on the cell as the membrane collapses, creating tensile stress on the chip [20]. At the same time, the cell wall becomes stiffer to keep its structure and resist changes in osmotic pressure [11,37]. These observations match our observations under 75 and 100 mmol/L NaCl treatment, including the generation of tensile stress and cell stiffening. Measurements of tobacco BY-2 cells under hyperosmotic stress also show that the resulting tensile stress makes the cells stiffer [38]. In contrast, under 125–150 mmol/L NaCl (Figure 8D), cellular Δ*S* no longer showed adaptive elevation but instead declined sharply, with the 150 mmol/L group exhibiting a steep drop. Additionally, *CVI* variations became significantly smaller and stabilized in later stages, while the actin cytoskeleton experienced rapid depolymerization followed by eventual network fragmentation. Cells showed complete plasmolysis and severe protoplast shrinkage. Together, these results suggest that high-intensity salt stress may exceed the cell’s mechanical regulatory capacity, leading to irreversible structural damage. Thus, the observed mechanical responses primarily reflect structural failure rather than adaptive regulation.

### 4.2. Actin Cytoskeleton Remodeling Underlies Cellular Mechanical Responses to Salt Stress

The actin cytoskeleton plays a pivotal role in maintaining cell morphology, regulating mechanical properties, and mediating mechanotransduction [7]. Consistent with the tensegrity model, actin filaments serve as the primary load-bearing elements under tensile stress, with their structural integrity governing global cellular mechanical equilibrium [39]. In this study, dynamic actin remodeling closely correlated with Δ*S* and *CVI* responses, underscoring its function as the structural foundation for salt stress-induced mechanical adaptations. At low-to-moderate NaCl concentrations, the actin cytoskeleton typically underwent a biphasic response: reversible bundling/thickening followed by partial depolymerization [36]. This phased restructuring aligned with the transient Δ*S* increase and *CVI* stiffening, suggesting that preserved cytoskeletal connectivity, particularly via Hechtian strands transmitting tensile forces [40,41], facilitated mechanical coupling between the plasma membrane and cell wall during plasmolysis. Such coordinated behavior likely generated the positive surface stress detected in DRPC measurements. Ketene et al. demonstrated that actin filaments play a primary role in cellular structural integrity and viscoelastic response; disrupting actin organization leads to a marked decrease in both cell elasticity and viscosity [42], providing direct causal evidence supporting our observation that microfilament depolymerization underlies the decline in Δ*S* and *CVI* at high NaCl concentrations. In contrast, under 150 mmol/L NaCl, the actin network underwent rapid disassembly, with fluorescence intensity dropping to near-background levels within minutes. Notably, this depolymerization exhibited a distinct temporal delay relative to plasmolysis, implying that actin depolymerization is not a direct consequence of membrane-wall detachment. Rather, cytoskeletal failure may reflect secondary damage from energy depletion, ion toxicity, and loss of mechanical homeostasis. Upon actin network disruption, tensile support and mechanosignaling capacity were abolished, culminating in synchronized Δ*S* and *CVI* anomalies.

### 4.3. Cellular Mechanical Parameters as Early Indicators of Salt-Stress Responses and Tolerance Thresholds

Numerous factors governing plant salt tolerance have been characterized, and the cellular and physiological responses to salt stress are well documented [43]. Upon salt exposure, plants rapidly perceive stress signals and initiate adaptive mechanisms. However, the transient nature of these signaling events often eludes detection by conventional physiological and biochemical assays. Unlike traditional biomarkers, cellular mechanical parameters exhibit distinct advantages, including rapid responsiveness and high integrative capacity. Furthermore, the methodology employed here enables real-time, continuous monitoring, thereby capturing dynamic stress responses. Our results demonstrate that under 75 to 100 mmol/L NaCl, Δ*S* and *CVI* changes precede the pronounced decline in microfilament fluorescence intensity, with a clear transitional phase occurring before irreversible morphological damage. This temporal precedence suggests that mechanical signals may function as early indicators of salt stress perception and regulatory adaptation. Notably, the concurrent shift in Δ*S* from negative to positive, accompanied by a transient increase in *CVI*, likely marks the transition from passive osmotic shrinkage to active mechanical resistance. This threshold response occurred at approximately 100 mmol/L NaCl, closely aligning with the established salt tolerance limit of these cells. These findings not only establish a quantitative framework for classifying salt stress severity but also introduce novel mechanophenotypic markers for screening salt-tolerant genotypes.

### 4.4. Advantages and Perspectives of DRPC in Plant Abiotic Stress Research

Double resonator piezoelectric cytometry (DRPC) enables label-free, non-destructive analysis, providing simultaneous resolution of surface stress and viscoelastic changes at the population scale, thereby overcoming spatial heterogeneity artifacts inherent in single-point measurements [22,23,24]. In this study, DRPC was systematically applied for the first time to investigate salt stress in tobacco cells, achieving multidimensional coupling analysis of mechanical parameters with cytoskeletal and morphological dynamics. These findings demonstrate the unique value of DRPC in plant abiotic stress research. It should be noted that DRPC measurements and confocal microscopy observations in this study employed different cell-supporting media: DRPC was performed in liquid suspension to ensure effective mechanical coupling between cells and the QCM sensor, whereas confocal imaging was conducted in a semi-solid matrix supplemented with 0.6 g/L phytagel to immobilize cells for high-resolution microfilament visualization. These distinct systems were dictated by their respective technical requirements and measurement objectives, forming a functionally complementary experimental strategy. Rather than direct numerical comparisons between the two systems, cross-validation was achieved through trend consistency analysis. The mechanical changes detected by DRPC in liquid culture and the structural alterations observed via confocal microscopy in gel-based systems exhibited high concordance under salt stress, further supporting the conclusion that salt stress induces coordinated mechanical and structural remodeling. Future work integrating DRPC with calcium signaling, reactive oxygen species (ROS) dynamics, cell wall composition analysis, and high-resolution cytoskeletal imaging could establish a unified mechanics-signaling-structure stress response model, enabling multi-scale correlative analysis from macroscopic mechanical responses to microscopic structural reorganization. Such advancements would not only enable rapid salt-tolerance phenotyping and molecular target screening but also offer novel technical strategies for crop stress-resilience breeding.

## 5. Conclusions

This study demonstrates a concentration-dependent relationship between NaCl stress and the structural integrity of microfilaments (F-actin) in tobacco cells, as well as the corresponding mechanical responses measured by double resonator piezoelectric cytometry (DRPC). At low NaCl concentrations (50 mmol/L), F-actin remained intact with only minor fluorescence attenuation, and cells exhibited reversible mechanical changes, including transient stiffening followed by recovery as indicated by *CVI*, and a mild “rise-and-fall” pattern in Δ*S*. Moderate NaCl stress (75–125 mmol/L) led to progressive F-actin depolymerization, accompanied by cytoskeletal disorganization and a shift in viscoelastic properties, characterized by initial stiffening (increased *CVI*) followed by partial recovery. Strikingly, this concentration range elicited adaptive mechanical responses, potentially indicating cell wall reinforcement as a defense mechanism. In contrast, high NaCl stress (150 mmol/L) resulted in irreversible F-actin depolymerization, complete cytoskeletal disassembly, and sustained mechanical failure, as demonstrated by a drastic Δ*S* decline, minimal *CVI* recovery, and severe plasmolysis. These results define a critical threshold (125–150 mmol/L NaCl) between reversible cytoskeletal remodeling and irreparable structural damage. By employing continuous dynamic monitoring, this study integrates multiparameter data from cellular mechanical measurements, fluorescence microscopy, and morphological observations, thereby systematically clarifying the dose-dependent effects of NaCl stress on tobacco suspension cells. Our findings delineate the core regulatory mechanisms underlying salt stress responses and provide critical cytological evidence for the coordinated regulation of plant adaptation to salinity. Moreover, this work underscores the utility of continuous multiparameter monitoring in studying dynamic biological processes. Future investigations could focus on leveraging early-warning biomarkers to develop rapid detection technologies, combined with molecular validation of actin cytoskeleton-mediated cell wall remodeling targets. Such efforts would facilitate theoretical and technological advancements in salt-tolerant crop breeding.

## Figures and Tables

**Table 1:** Mean and standard deviations of osmotic pressures in tobacco culture media with different concentrations of NaCl added (mean ± SD, *n* = 3).

NaCl (mmol/L)	Average Osmotic Pressure (mOsm·kg^−1^)	Standard Deviation (s)
0	203.67	0.58
50	303.33	2.31
75	353.67	1.15
100	390.00	1.00
125	431.67	1.15
150	464.67	0.58

## Data Availability

The published article includes all datasets generated or analyzed during this study. The authors will be happy to share raw data on request.
